# Evaluation of the Potential Sensitization of Chlorogenic Acid: A Meta-Analysis

**DOI:** 10.1155/2013/208467

**Published:** 2013-05-16

**Authors:** Mingbao Lin, Wan Gong, Qian Chen, Lijuan Sun, Yingchao Wang, Xiaohui Fan

**Affiliations:** Pharmaceutical Informatics Institute, College of Pharmaceutical Sciences, Zhejiang University, 866 Yuhangtang Road, Hangzhou 310058, China

## Abstract

Chlorogenic acid (CGA) widely exists in many plants, which are used as medicinal substances in traditional Chinese medicine injectables (TCMIs) that have been widely applied in clinical treatments. However, it is still controversial whether CGA is responsible for TCMIs-related hypersensitivity. Several studies have been performed to evaluate its potential sensitization property, but the results were inconclusive. Therefore, the aim of this study was to evaluate its potential sensitization systematically using meta-analysis based on data extracted from literatures, searching databases of PubMed, EMBASE, ISI Web of Knowledge, CNKI, VIP, and CHINAINFO from January 1979 to October 2012, a total of 108 articles were retrieved by electronic search strategy, out of which 13 studies met the inclusion criteria. In ASA test, odds ratio of behavior changes was 4.33 (1.62, 11.60), showing significant changes after CGA treatment (*P* = 0.004). Serum IgG, serum histamine, PLN cellularity, and IgG1 AFCs were significantly enhanced after CGA treatment (*P* < 0.05). Totally, these results indicated that CGA could induce a positive reaction in potential sensitization, and intravenous administration of it might be a key factor for sensitization triggering, which could at least warrant more careful application of TCMIs containing CGA in clinical practices.

## 1. Introduction

Chlorogenic acid (CGA), an easter of caffeic and quinic acid from the phenylpropanoid metabolism in many plants [1, 2], widely exists in many Chinese herbal medicines, such as *Flos Lonicerae Japonicae* [3], *Houttuynia cordata* [4], and *Lonicera japonica* Thumb [5]. These herbal medicines are frequently used as medicinal substances in traditional chinese medicine injectables (TCMIs), which have played an important role in clinical treatment of many diseases, such as cardiovascular and cerebrovascular diseases, viral diseases, and cancer. It has been demonstrated that CGA has various potential therapeutic biological activities, including anti-inflammatory activity [6], antioxidation activity [7], antiviruses activity [8], antitumor activity [9], and neuroprotective effects [10]. 

However, since the 1960s, it has been remained considerable controversial whether CGA has potential sensitization activity, which is known to be associated with systematic safety [11–13]. With the wide application in China, the safety issues of TCMIs are becoming increasingly critical in recent years. One research indicated that TCMIs accounted for 66.7% of adverse reactions in traditional Chinese medicines [14]. Furthermore, it is estimated that 90% of these adverse reactions, especially the systemic safety of sensitization, are related to TCMIs containing CGA [15]. As a consequence, CGA has been considered to be a major chemical ingredient leading to allergic reactions by most clinicians. However, with respect to several studies performed for potential sensitization evaluation of CGA or TCMIs containing it, not all outcomes are clear-cut but consistency seems to be a big problem [16, 17]. Consequently, more studies need to be done to prove that CGA is the chief culprit of TCMIs to induce potential sensitization in clinics.

Randomized animal studies represent the best study designs for potential sensitization of CGA evaluating. However, with the discrepancy in experimental designs, different investigators could reach disparate outcomes and diverse conclusions. A systematic literature analysis may address this problem from general points of view by allowing integration of existing information, then providing messages for rational decision making [18]. The systematic literature analysis had been done to give compelling evidences for hydrocortisone treatment of the neonatal premature death, which have saved ten thousand of lives of premature babies [19]. As an effective systematic literature analysis tool, meta-analysis has previously been used for the optimization of experimental animal models and design of clinical trials improvement [20, 21]. Meta-analysis on animal studies could also optimize many valuable indicators to make rational decisions and draw proper conclusions [22]. Moreover, it can establish whether experimental findings are consistent, reduce bias, and improve reliability and accuracy of conclusions with quality criteria having been fulfilled [23]. 

Therefore, in response to the controversies of published studies on the potential sensitization of CGA, meta-analysis was set out to provide a complete and systematic overview of all the literatures available, give an insight into the quality of literatures in the field, and also supply reliable information for clinical practice of TCMIs containing CGA. The PRISMA (Preferred Reporting Items for Systematic Reviews and Meta-Analyses) statement was used to help guide reporting of this meta-analysis [24].

## 2. Methods

### 2.1. Literature Search Strategy

According to the Cochrane review methodology, a systematic computerized searching for published articles was carried out by using “Themes” combining with “Keywords” from January 1979 to October 2012 on the potential sensitization of CGA assessment in databases of PubMed, EMBASE, ISI Web of Knowledge, CNKI, VIP, and CHINAINFO. Languages were restricted to Chinese and English. The full search strategies for Pubmed, EMBASE, ISI Web of Knowledge are “chlorogenic acid,” “allergy,” “sensitization,” and “hypersensitivity”; and for CNKI, VIP, and CHINAINFO included in the following search components: “*绿原酸*”, “*过敏*”, “*致敏*”, and “*变态反应*” in Chinese.

### 2.2. Literature Inclusion and Exclusion Criteria

Studies were included in the systematic assessments if they fulfilled all of the following criteria: (1) studies were performed in mice, rats, or guinea pigs *in vivo*; (2) studies conformed to the principle of randomized controlled design; (3) methods and criteria of evaluation were basically consistent with Technology Guidelines on Studies for the Immune Toxicities (Allergic and Light Allergic) in Chinese Medicine and Natural Medicine, and the evaluating indicators should contain active systemic anaphylaxis (ASA), passive cutaneous anaphylaxis (PCA) and so on, and (4) the published format for studies were original full papers which presented unique data, including average, standard deviation, and animal numbers.

Studies were excluded if (1) they were performed *in vitro* or *ex vivo*; (2) the principles of randomized controlled study design were not conformed; (3) methods and criteria of evaluation did not agree with Technology Guidelines on Studies for the Immune Toxicities (Allergic and Light Allergic) in Chinese Medicine and Natural Medicine; (4) data was not complete, such as the animal numbers, and without full text obtained; and (5) results have been published in review articles.

### 2.3. Study Characteristics and Data Extraction

The study characteristics and data items were extracted from the included articles, such as animal species, stain, sex, animal numbers of treatment and control groups, measures of randomization, numbers of excluded animals in statistical analysis, and outcome measures and results reported. In addition, bibliographic details, including author, journal, and year of publication were also registered. Serum IgE, IgG, and histamine, behavior change in ASA, PLN cellularity, and IgG1 antibody forming cells (AFCs) in popliteal lymph node assay (PLNA), and blue skin patch test in PCA were assessed. For quantitative data, raw data or group averages, standard deviation (SD) or standard error (SE), and animal numbers per group (*n*) were extracted from the published results. As for qualitative data, results of positive animal numbers of each group were taken from the papers for the analysis. If there were two or more identical groups in qualitative data, the data would be pooled. If several outcomes were measured at various time points, the time point with the greatest efficacy was chosen.

### 2.4. Assessment of Methodological Quality

The methodological quality of the included studies was evaluated by applying a literature quality 10-item checklist derived from Critical Appraisal Skills Programme (CASP), published by Britain Evidence-Based Medicine Center, which applied a systematic approach to a assess study validity, methodological quality and external validity [25]. Consistent with current guidelines, low-quality studies did not weighed by quality scores and were not excluded, which can only help readers have a general idea of the quality of studies. Study quality was scored independently by two reviewers, and the discrepancies were clarified by discussion with a third investigator.

### 2.5. Data Synthesis and Statistical Analyses

Data of the included studies were synthesized and analyzed using Review Manager 5.1 (Copenhagen, The Cochrane Collaboration, 2011). Meta-analysis was performed for the quantitative continuous outcome measures of serum IgE, IgG, and histamine, PLN cellularity, and IgG1 AFCs, by computing the weighted means difference (WMD) and 95% confidence interval (CI) for normal distribution of the extracted data, while standard. mean difference (SMD) and 95% confidence interval (CI) for nonnormal distribution. For the outcomes of behavior changes of ASA, odds ratio (OR) and 95% CI were calculated. Results extracted from the comparable studies were pooled using fixed and random effects models. The *Q*-test for homogeneity was considered to be statistically significant when *P* < 0.05, which cast doubt on the statistical validity of the synthesis. Random effect models were selected for data pooling as *P* < 0.05 in *Q*-test; on the contrary, fixed effect models were chosen to evaluate the sensitivity of our results. In addition, the possibility of publication bias for ASA outcome measure was evaluated by visually evaluating the possible asymmetry in funnel plots.

## 3. Results

### 3.1. Study Selection

**Figure 1:**
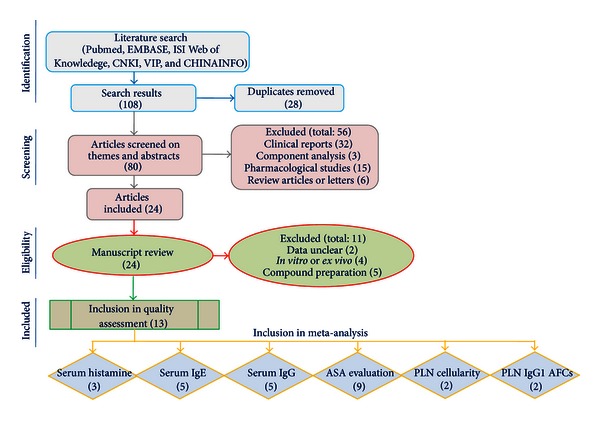
Flow chart of study selection. The number of studies in each phase is indicated between brackets.

108 records, including 19 English and 89 Chinese articles, were retrieved by electronic search strategy from databases of Pubmed, EMBASE, ISI Web of Knowledge, CNKI, VIP, and CHINAINFO. Among them, 80 records were found to be unique after removing duplicates, and 24 papers met our inclusion criteria by the theme and abstract analysis. Finally, 13 articles, including 3 English and 10 Chinese articles, were retrieved on the basis of predefined criteria by full texts reading (see Figure 1).

### 3.2. Study Characteristics and Quality Assessment

The characteristics of the included studies were summarized in Table 1, including animal species and numbers, research methods, indicators, and conclusion. Since the 1970s, there were very few articles on the subject of potential sensitization by CGA in English; however, it attracted much attention after the year of 2000 in China. Therefore, the majority of retrieved studies were published after 2000.

The results of the quality assessment of the 13 included studies in this systematic analysis were shown in Table 1. On average, the score of the reports quality was 14.5. In the quality assessment, only 5 of the 13 animal studies reported randomization of the animals across treatment groups; moreover, no study reported blind measurements for any other outcome and the number of animals being absent. The results showed that the quality of these included studies was not high but in a medium quality.

### 3.3. Meta-Analysis of Outcome Measures of Sera **IgE*, *IgG*, and *Histamine**

**Figure 2:**
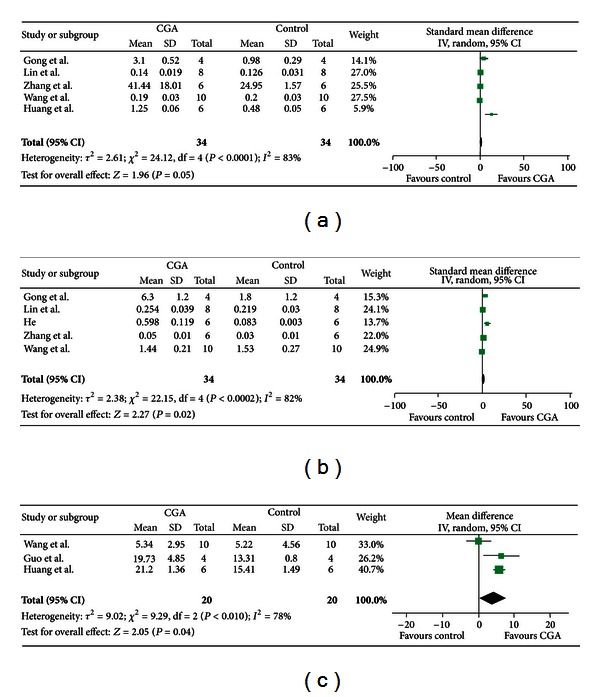
Meta-analysis of the indicators of sera IgE, IgG, and histamine. (a) The result of combined effects of serum IgE; (b) the result of combined effects of serum IgG; and (c) the result of combined effects of serum histamine.

Results for the serum-related indicators of IgE, IgG, and histamine were summarized in Figure 2. Except histamine (WMD and 95% CI), CGA significantly induced alterations in these indicators based on the SMD and 95% CI. As shown in Figure 2(a), five studies were included, among which two reported a negative effect of CGA on serum IgE. Random effect models were chosen for meta-analysis of serum IgE since there was a significant difference in heterogeneity test (*χ* ^2^ = 24.12, *P* < 0.0001). Overall analysis of serum IgE indicated that there was no significant difference in CGA-induced serum IgE formation (SMD = 1.73, 95% CI (0.00–3.45), *P* = 0.05). 

According to heterogeneity test results of serum IgG (*χ* ^2^ = 22.15, *P* = 0.0002), there was a significance variance in serum IgG formation induced by CGA (SMD = 1.82, 95% CI (0.25–3.39), *P* = 0.02) using random effect models (Figure 2(b)). 

Meta-analysis of serum histamine was shown in Figure 2(c); since significant difference existed in heterogeneity test (*χ* ^2^ = 9.29, *P* < 0.01), random effect models were used for this indicator. Overall analysis of serum histamine showed that its level was significantly induced by CGA treatment (WMD = 4.08, 95% CI (0.19–7.98), and *P* = 0.04). 

In addition to assessing the robustness of these results, sensitivity analysis was also undertaken by exchange of random effect models with fixed effect models; the results showed that the selection of methods did not significantly alter the outcomes of each indicator (data not shown). 

### 3.4. Meta-Analysis of Outcome Measures of the Behavior Changes in ASA

**Figure 3:**
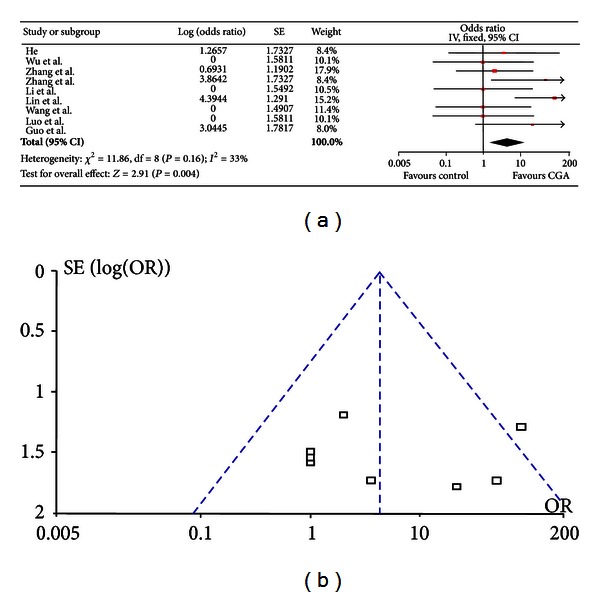
Meta-analysis of the behavior change in ASA. (a) Combined effects after CGA treatment; (b) the funnel plot for literature publication bias observation.

Logarithmic of odds ratio (log (odds ratio)) and SE were transformed from animal numbers of positive and negative reactions in behavior changes. As results of positive reaction being “zero” in both CGA treatment and control group, a fixed value—“one” was set for further analysis [18, 37]. Results of the behavior changes in meta-analysis were shown in Figure 3(a). A total of nine articles were included and four articles reported a negative effect of CGA on animal behaviors. Since there was no significant homogeneity observed in these included studies (*χ* ^2^ = 11.86, *P* = 0.16), the fixed effect models were chosen to pool effects of behavior changes in ASA. Results of overall analysis indicated that animal behaviors could be significantly changed after CGA treatment (OR = 4.33, 95% CI (1.62–11.60), *P* = 0.004). In addition, as showed in Figure 3(b), there was no obvious literature publication bias in these included studies by visually evaluation of the funnel plots. Sensitivity analysis was also assessed by using exchange of fixed models with random effect models and the consistent results suggested that sensitivity of the model in the indicator of behavior change was favorable (data not shown).

### 3.5. Meta-Analysis of Outcome Measures of PLN Cellularity and IgG1 AFCs

**Figure 4:**
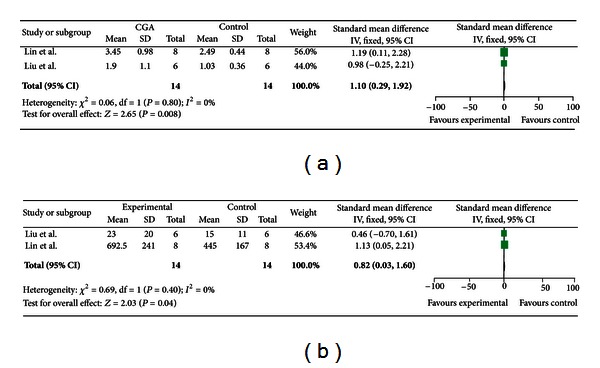
Meta-analysis of PLN cellularity and IgG1 AFCs. (a) The result of combined effects of PLN cellularity; (b) the result of combined effects of PLN IgG1 AFCs.

A total of two articles were included in the evaluation of CGA sensitization by PLNA; however, the opposite results were obtained from these two. Results for the PLNA-related indicators of PLN cellularity and IgG1 AFCs were summarized in Figure 4. As shown in Figure 4(a), fixed effect models were chosen for meta analysis of PLN cellularity due to a significant difference in heterogeneity test (*χ* ^2^ = 0.06, *P* = 0.8). The results of overall analysis showed that CGA could significantly increase PLN cellularity (SMD = 1.10, 95% CI (0.29–1.92), *P* = 0.008). 

Mata-analysis of IgG1 AFCs was showed in Figure 4(b). Fixed effect models were chosen according to heterogeneity test results (*χ* ^2^ = 0.69, *P* = 0.40). The results of overall analysis demonstrated that there was a significance in IgG1 AFCs induced by CGA [SMD = 0.82, 95% CI (0.03–1.60), *P* = 0.04].

### 3.6. Analysis of the PCA Results of Included Studies

Data of PCA from five included studies were summarized in Table 2. Using the standards of determination, negative results were extracted from all included studies. Therefore, meta-analysis was not performed in PCA.

## 4. Discussions

From individual randomized studies, it is still unclear and controversial whether CGA might induce potential sensitization after its treatment. To shed light on the disagreement on this issue, meta-analysis is employed to analyze different independent studies with the same purpose. Our hypothesis for this study is that a systematic analysis is the best approach to quantitatively show a pooled result for the potential sensitization of CGA and examine the bias of the published literature at the same time. For this purpose, 13 studies were retrieved to summarize and analyze systematically in this study, and a positive reaction on potential sensitization of CGA was identified by meta-analysis. Additionally, the quality of included studies was evaluated by CASP system and a favorable quality was shown for these studies.

According to The Chemical Drug Excitant, Irritability and Hemolytic Research Technology Guideline approved by SFDA of China, and The Traditional Chinese Medicine Injection Safety Inspection Application Guideline in appendix of Pharmacopoeia of the People's Republic of China (2010 edition), ASA and PCA should be performed to validate the sensitization of TCMIs [38, 39]. Although there are lots of limitations in the behavioral changes in ASA, such as subjective judgments in the changes of symptoms and difficult quantification of its systematical descriptions to the character and strength of immune reactions, it is still the key method with great importance for the evaluation of the potential sensitization currently. Up to now, the indicator of behavioral changes is still an irreplaceable evaluation index of anaphylaxis preclinical. According to inclusion criteria, a total of 9 studies on behavioral changes in ASA were included in present study and pooled analysis were performed by meta-analysis. Our results suggested that animal behaviors could be significantly altered after CGA treatment, and no significance was shown in the literature publishment bias.

However, with regard to the PCA results of included studies, all negative reactions were observed after CGA stimulating, so that PCA test may not be suitable for potential sensitization evaluation of small molecules, such as CGA. There are several possible explanations, such as sensitivity limitations of PCA skin tests, difference between subcutaneous exposure and conventional exposure routes (orally or intravenously treated), and lack of metabolism or combing macromoleculeswithsubcutaneously treated to change hapten to antigen *in vivo* [28]. 

To overcome the limitations in ASA and PCA test, serum indicators can make it up to some extent, such as histamine, IgE antibody, and IgG antibody, which have been proved to be correlated with the type I hypersensitivity [40, 41]. In this study, the pooled results from meta-analysis showed that CGA might significantly induce serum IgG and histamine formation in ASA animal model. The results suggested that CGAmight induce initiative immune reactions systemically. In addition, we observed a negative results for serum IgE after treated by CGA but a clear trend of increased level was obtained (*P* = 0.05), which probably correlated with the limitations of literature numbers and heterogeneity in these literatures. 

PLNA, a reliable, fast, and reproducible animal experiment for IDHR assessment [42–47], has been used for the identification of numerous drugs and chemicals known to induce IDHR in humans, such as penicillin, diclofenac, and cadmium [44, 46, 48–50], which can effectively augment the low sensitivity shortcoming of serum indicators in immune activation. In this work, two studies were included in meta-analysis of PLNA parameters; one proved a positive reaction by using an intravenous mouse model [14, 35], while another showed a negative result by subcutaneous administration. The inconsistent results of PLN cellularity and IgG1 AFCs were pooled by meta-analysis and the pooled results showed that CGA might significantly increase PLN cellularity and induce IgG1 AFCs formation. The results further suggested the potential of CGA to systemically initiate immune reactions. And the discrepancy of the results may be correlated with the routes of administration, indicating that CGA easily triggers allergic reactions by intravenous administration.

In conclusion, the results from overall meta-analysis indicated that a positive reaction of potential sensitization could be induced by CGA. To prevent further allergic adverse complications, such findings from our work may at least warrant more careful evaluation in the application of CGA in clinical practice, especially for TCMIs containing it. To note, interactions between ingredients of TCMIs may also be involved in sensitizations of TCMIs, which was not covered in current work and clearly needs to be further explored. However, this conclusion must be moderated by the fact that many confounding influent factors exist, such as limitations of literature numbers. Despite the inherent limitations in this meta-analysis, it has presented the best outcomes in the literature. There is a crucial need for more high-quality studies in this field and for a more sensitive experimental model applicable to evaluate the potential sensitization of TCMIs to make an evidence-based decision.

## Supplementary Material

Appendix S1 PRISMA 2009 Checklist.
Appendix S2 Full search strategy for PubMed.

## Figures and Tables

**Table 1:** Characteristics and quality of included studies.

Study	Year	Language	Animal	Sample (T/C)	Route of AD^e^	Duration (day)	Method	Outcome measure	Conclusion	CASP score
Wang et al. [16]	2011	Chinese	GP^a^	10/10	ip, iv	14	ASA	IgE, IgG, His^b^, BC^c^	Negative	14
Zhang et al. [17]	2011	Chinese	GP	6/6	ip, iv	21	ASA, PCA	BC, BSPT^d^	Negative	16
Lin et al. [26]	2010	Chinese	Rat, GP	19/11	ip, iv	13	ASA, PCA	BC, BSPT	Positive	13
Guo et al. [27]	2011	Chinese	BN rat, GP	4/4	ip, iv	14	ASA	His, BC	Positive	15
Huang et al. [28]	2010	Chinese	GP	6/6	ip, iv	14	ASA	IgE, IgG	Positive	16
Luo et al. [29]	2009	Chinese	Rat, GP	5/5	ip, iv	8	ASA, PCA	BC, BSPT	Negative	13
He et al. [30]	2011	Chinese	Mouse, GP	6/6	iv	14	ASA	IgG, BC	Positive	14
Wu et al. [31]	2010	Chinese	GP	5/5	ip, iv	10	ASA, PCA	BC, BSPT	Negative	14
Li et al. [32]	2008	Chinese	Rat, GP	6/6	ip, iv	14	ASA, PCA	BC, BSPT	Positive	15
Zhang et al. [33]	2010	Chinese	BN rat	6/6	iv	13	ASA	IgE, IgG, BC	Positive	15
Gong et al. [34]	2004	English	mouse	4/4	ip, in	1	ASA	IgE, IgG	Positive	13
Lin et al. [35]	2012	English	Mouse	8/8	iv	11	PLNA	Cellularity, IgG1 AFCs	Positive	16
Liu et al. [36]	2010	English	Mouse	6/6	si	7	PLNA	Cellularity, IgG1 AFCs	Negative	15

^a^Guinea pig; ^b^histamine; ^c^behavior change; ^d^blue skin patch test; ^e^administration; ip: intraperitoneally; in: intranasally; iv: intravenously; and si: subcutaneous injection.

**Table 2:** Results of PCA evaluation of included studies.

Study or subgroup	Results of PCA	Standards of determination
Wu et al. [31]	Negative	Positive: diameter ≥5 mm
Zhang et al. [17]	Negative	
Li et al. [32]	Negative	
Lin et al. [26]	Negative	
Luo et al. [29]	Negative	

## References

[1] Mølgaard P, Ravn H (1988). Evolutionary aspects of caffeoyl ester distribution in Dicotyledons. *Phytochemistry*.

[2] de Moura SA, Negri G, Salatino A (2011). Aqueous extract of brazilian green propolis: primary components, evaluation of inflammation and wound healing by using subcutaneous implanted sponges. *Evidence-Based Complementary and Alternative Medicine*.

[3] Tang D, Li HJ, Chen J, Guo CW, Li P (2008). Rapid and simple method for screening of natural antioxidants from Chinese herb Flos Lonicerae Japonicae by DPPH-HPLC-DAD-TOF/MS. *Journal of Separation Science*.

[4] Nuengchamnong N, Krittasilp K, Ingkaninan K (2009). Rapid screening and identification of antioxidants in aqueous extracts of Houttuynia cordata using LC-ESI-MS coupled with DPPH assay. *Food Chemistry*.

[5] Chen Z, Zhang J, Chen G (2008). Simultaneous determination of flavones and phenolic acids in the leaves of Ricinus communis Linn. by capillary electrophoresis with amperometric detection. *Journal of Chromatography B: Analytical Technologies in the Biomedical and Life Sciences*.

[6] dos Santos MD, Almeida MC, Lopes NP, de Souza GEP (2006). Evaluation of the anti-inflammatory, analgesic and antipyretic activities of the natural polyphenol chlorogenic acid. *Biological and Pharmaceutical Bulletin*.

[7] Kaur R, Sharma U, Singh B, Arora S (2011). Antimutagenic and antioxidant characteristics of Chukrasia tabularis a Juss extracts. *International Journal of Toxicology*.

[8] Lee JH, Park JH, Kim YS, Han Y (2008). Chlorogenic acid, a polyphenolic compound, treats mice with septic arthritis caused by Candida albicans. *International Immunopharmacology*.

[9] Jin UH, Lee JY, Kang SK (2005). A phenolic compound, 5-caffeoylquinic acid (chlorogenic acid), is a new type and strong matrix metalloproteinase-9 inhibitor: Isolation and identification from methanol extract of Euonymus alatus. *Life Sciences*.

[10] Huang SM, Chuang HC, Wu CH, Yen GC (2008). Cytoprotective effects of phenolic acids on methylglyoxal-induced apoptosis in Neuro-2A cells. *Molecular Nutrition & Food Research*.

[11] Layton LL, Greene FC, Panzani R (1965). Allergy to green coffee. Failure of patients allergic to green coffee to react to chlorogenic acid, roasted coffee, or orange. *Journal of Allergy*.

[12] Freedman SO, Shulman R, Krupey J, Sehon AH (1964). Antigenic properties of chlorogenic acid. *Journal of Allergy*.

[13] Freedman SO, Siddiqi AI, Krupey J, Sehon AH (1962). Identification of a simple chemical compound (chlorogenic acid) as an allergen in plant materials causing human atopic disease. *Transactions of the Association of American Physicians*.

[14] Lin M, Sun W, Wang Y (2012). An intravenous exposure mouse model for prediction of potential drug-sensitization using reporter antigens popliteal lymph node assay. *Journal of Applied Toxicology*.

[15] Li Q, Zhang XY, Chen GS (2009). Adverse effect and mechanism of chlorogenic acid in clearing heat and detoxication traditional Chinese medicine injections. *Chinese Journal of Modern Applied Pharmacy*.

[16] Wang ZG, Wang DQ, Yu YH (2011). Study on allergenicity of chlorogenic acid in Qingkailing injection. *China Journal of Chinese Materia Medica*.

[17] Zhang XY, Chen AJ, LI Q (2011). Experimental study on chlorogenic acid allergy by passive cutaneous anaphylaxis test and active systemic anaphylaxis test. *Chinese Journal of Traditional Medical Science and Technology*.

[18] Nizard RS, Biau D, Porcher R (2005). A meta-analysis of patellar replacement in total knee arthroplasty. *Clinical Orthopaedics and Related Research*.

[19] Carnathan GW, Metcalf LE, Cochrane RL, Nutting EF, Black DL (1987). Relationship between progesterone suppression and pregnancy in rats. *The Journal of Pharmacy and Pharmacology*.

[20] Pound P, Ebrahim S, Sandercock P, Bracken MB, Roberts I (2004). Where is the evidence that animal research benefits humans?. *British Medical Journal*.

[21] van der Worp HB, Sena ES, Donnan GA, Howells DW, Macleod MR (2007). Hypothermia in animal models of acute ischaemic stroke: a systematic review and meta-analysis. *Brain*.

[22] Wever KE, Menting TP, Rovers M (2012). Ischemic preconditioning in the animal kidney, a systematic review and meta-analysis. *Plos One*.

[23] Handoll HHG, Gillespie LD, Gillespie WJ (2004). Methodologic issues in systematic reviews and meta-analyses. *Clinical Orthopaedics and Related Research*.

[24] Moher D, Liberati A, Tetzlaff J (2009). Preferred reporting items for systematic reviews and meta-analyses: the PRISMA statement. *PLOS Medicine*.

[25] Center BE-BM Critical Appraisal Skills Programme.

[26] Lin M, Qian HQ, Zhang T (2010). Experimental study on allergic reactions caused by chlorogenic acid. *Shanghai Journal of Traditional Chinese Medicine*.

[27] Guo SS, Wang YZ, Jin YH (2011). Establishment and applicability evaluation of animalmodel which was suitable to evaluate immediate hypersensitivityinduced by injections of traditional Chinese medicine in BN rats. *China Journal of Chinese Materia Medica*.

[28] Huang XW, Liao HB, Liu P (2010). Study on sensitization and mechanism of CGA-BSA. *China Journal of Chinese Materia Medica*.

[29] Luo F, Bao X, Lin DS (2009). Allergic study of chlorogenic acid to animal. *West China Journal of Pharmaceutical Sciences*.

[30] He Z (2011). *Association Study on Allergic Reaction Between Chlorogenic Acid and Qingkailing Injection*.

[31] Wu XD, Yang HR, Lin DS (2010). Comprehensive research and evaluation of chlorogenic acid allergy. *China Journal of Chinese Materia Medica*.

[32] Li JF, Li Y, Chen Q (2008). Research of the immunotoxicity of Shuanghuanglian injection. *Traditional Chinese Drug Research & Clinical Pharmacology*.

[33] Zhang RX, Tang NP, Lin HX (2010). Allergenicity assessment and comparison between chlorogenic acid and Shuanghuanglian Fenzhenji. *Modernization of Traditional Chinese Medicine and Materia Medica, World Science and Technology*.

[34] Gong J, Liu FT, Chen SS (2004). Polyphenolic antioxidants enhance IgE production. *Immunological Investigations*.

[35] Lin M, Gong W, Wang Y (2012). Structure-activity differences of chlorogenic acid and its isomers on sensitization via intravenous exposure. *International Journal of Toxicology*.

[36] Liu Z, Liu Z, Shi Y, Zhou G (2010). Evaluation of the immunosensitizing potential of chlorogenic acid using a popliteal lymph node assay in BALB/c mice. *Food and Chemical Toxicology*.

[37] Friedrich JO, Adhikari NKJ, Beyene J (2007). Inclusion of zero total event trials in meta-analyses maintains analytic consistency and incorporates all available data. *BMC Medical Research Methodology*.

[38] Commission SP (2010). *2010 Edition of the People's Republic of China Pharmacopoeia*.

[39] SFDA The chemical drug excitant, irritability and hemolytic research technology guideline.

[40] Bouike G, Nishitani Y, Shiomi H (2011). Oral treatment with extract of Agaricus blazei Murill enhanced Th1 response through intestinal epithelial cells and suppressed OVA-sensitized allergy in mice. *Evidence-Based Complementary and Alternative Medicine*.

[41] Frew A (2011). General principles of investigating and managing drug allergy. *British Journal of Clinical Pharmacology*.

[42] Kammuller ME, Thomas C, de Bakker JM, Bloksma N, Seinen W (1989). The popliteal lymph node assay in mice to screen for the immune disregulating potential of chemicals—a preliminary study. *International Journal of Immunopharmacology*.

[43] Gehrs BC, Smialowicz RJ (1999). Persistent suppression of delayed-type hypersensitivity in adult F344 rats after perinatal exposure to 2,3,7,8-tetrachlorodibenzo-p-dioxin. *Toxicology*.

[44] Gutting BW, Updyke LW, Amacher DE (2002). Diclofenac activates T cells in the direct popliteal lymph node assay and selectively induces IgG1 and IgE against co-injected TNP-OVA. *Toxicology Letters*.

[45] Nierkens S, Aalbers M, Bol M (2005). Differential requirement for CD28/CTLA-4-CD80/CD86 interactions in drug-induced type 1 and type 2 immune responses to trinitrophenyl-ovalbumin. *Journal of Immunology*.

[46] Carey JB, Allshire A, van Pelt FN (2006). Immune modulation by cadmium and lead in the acute reporter antigen-popliteal lymph node assay. *Toxicological Sciences*.

[47] Løvik M, Alberg T, Nygaard UC, Samuelsen M, Groeng EC, Gaarder PI (2007). Popliteal lymph node (PLN) assay to study adjuvant effects on respiratory allergy. *Methods*.

[48] Gleichmann E, Gleichmann H (1976). Graft versus host reaction: a pathogenetic principle for the development of drug allergy, autoimmunity, and malignant lymphoma in non chimeric individuals. Hypothesis. *The Journal of Cancer Research and Clinical Oncology*.

[49] Aida T, Kimura T, Ishikawa N, Shinkai K (1998). Evaluation of allergenic potential of low-molecular compounds by mouse popliteal lymph node assay. *Journal of Toxicological Sciences*.

[50] Samuelsen M, Nygaard UC, Løvik M (2008). Allergy adjuvant effect of particles from wood smoke and road traffic. *Toxicology*.

